# Crystal structure of the C-terminal globular domain of the third paralog of the *Archaeoglobus fulgidus* oligosaccharyltransferases

**DOI:** 10.1186/1472-6807-13-11

**Published:** 2013-07-01

**Authors:** Shunsuke Matsumoto, Atsushi Shimada, Daisuke Kohda

**Affiliations:** 1 Division of Structural Biology, Medical Institute of Bioregulation, Kyushu University, Maidashi 3-1-1, Higashi-ku, Fukuoka, Japan; 2 RIKEN Systems and Structural Biology Center, Suehiro-cho 1-7-22, Tsurumi, Yokohama, Japan; 3 Research Center for Advanced Immunology, Medical Institute of Bioregulation, Kyushu University, Maidashi 3-1-1, Higashi-ku, Fukuoka, Japan

**Keywords:** AglB, *Archaeoglobus fulgidus*, Crystal structure, N-glycosylation, MBP fusion, Oligosaccharyltransferase

## Abstract

**Background:**

Protein N-glycosylation occurs in the three domains of life. Oligosaccharyltransferase (OST) transfers an oligosaccharide chain to the asparagine residue in the N-glycosylation sequons. The catalytic subunits of the OST enzyme are STT3 in eukaryotes, AglB in archaea and PglB in eubacteria. The genome of a hyperthermophilic archaeon, *Archaeoglobus fulgidus*, encodes three paralogous AglB proteins. We previously solved the crystal structures of the C-terminal globular domains of two paralogs, AglB-S*hort* 1 and AglB-S*hort* 2.

**Results:**

We determined the crystal structure of the C-terminal globular domain of the third AglB paralog, AglB-L*ong*, at 1.9 Å resolutions. The crystallization of the fusion protein with maltose binding protein (MBP) afforded high quality protein crystals. Two MBP-AglB-L molecules formed a swapped dimer in the crystal. Since the fusion protein behaved as a monomer upon gel filtration, we reconstituted the monomer structure from the swapped dimer by exchanging the swapped segments. The C-terminal domain of *A. fulgidus* AglB-L includes a structural unit common to AglB-S1 and AglB-S2. This structural unit contains the evolutionally conserved WWDYG and DK motifs. The present structure revealed that *A. fulgidus* AglB-L contained a variant type of the DK motif with a short insertion, and confirmed that the second signature residue, Lys, of the DK motif participates in the formation of a pocket that binds to the serine and threonine residues at the +2 position of the N-glycosylation sequon.

**Conclusions:**

The structure of *A. fulgidus* AglB-L, together with the two previously solved structures of AglB-S1 and AglB-S2, provides a complete overview of the three AglB paralogs encoded in the *A. fulgidus* genome. All three AglBs contain a variant type of the DK motif. This finding supports a previously proposed rule: The STT3/AglB/PglB paralogs in one organism always contain the same type of Ser/Thr-binding pocket. The present structure will be useful as a search model for molecular replacement in the structural determination of the full-length *A. fulgidus* AglB-L.

## Background

Asparagine-linked glycosylation (N-glycosylation) of proteins is the most ubiquitous post-translational modification in eukaryotes, all archaea, and some eubacteria [1,2]. Oligosaccharyltransferase (OST) catalyzes the transfer of an oligosaccharide chain from a lipid-linked oligosaccharide (LLO) donor to the asparagine residues in the N-glycosylation sequon, Asn-X-Ser/Thr (X ≠ Pro) [3,4]. In higher eukaryotes, OST is a multi-subunit and membrane-associated protein complex, whereas the OSTs from lower eukaryotes, archaea and eubacteria are single-subunit membrane proteins [5,6]. The catalytic subunit of the OST enzyme is the only subunit conserved evolutionally across the three domains of life, and it is referred to as STT3 (Staurosporine and Temperature sensitivity 3) in eukaryotes, AglB (Archaeal Glycosylation B) in archaea, and PglB (Protein Glycosylation B) in eubacteria. The STT3/AglB/PglB proteins share a common overall architecture, consisting of an N-terminal multi-spanning transmembrane region and a C-terminal globular domain [7-9]. Despite the very low overall sequence identity, multiple sequence alignments revealed a few short conserved motifs: two diacidic DXD motifs in the N-terminal transmembrane region, and a well-conserved 5-residue WWDYG motif in the C-terminal globular domain [10-12]. We previously determined the crystal structures of the C-terminal globular domains of four AglB proteins and one PglB protein [13-16]. The structural comparison revealed the common structural unit and the unique structural units specific to each protein. In addition, structure-aided sequence alignment led to the discovery of new short motifs, the DK and MI motifs, based on the fact that the two motifs are located at spatially equivalent positions close to the WWDYG motif [14]. The consensus sequences of the DK and MI motifs are D*XX*K*XXX*(M/I) and M*XX*I*XXX*(I/V/W), respectively, where *X* means any amino acid residue. Since the side chains of the signature residues of the two motifs have very different chemical properties (i.e., D↔M and K↔I), the identification of the new motifs would have been almost impossible without reference to the three-dimensional structures.

In 2011, the crystal structure of full-length *Campylobacter lari* PglB, in a complex with an acceptor peptide, was reported at 3.4 Å resolutions [17]. This structure revealed several important features of the STT3/AglB/PglB protein, including 1) the catalytically important acidic residues and a divalent metal ion in the transmembrane region, 2) the putative amide nitrogen activation mechanism of the side chain of the acceptor asparagine residue, and 3) the binding pocket in the C-terminal globular domain that recognizes the serine and threonine residues at the +2 position in the N-glycosylation sequon. The locations of the short amino acid motifs seem to correspond well with these functionally important structures. The conserved acidic residues in the two DXD motifs are involved in divalent ion coordination and amide nitrogen activation. Trp-Trp-Asp part of the conserved WWDYG motif and the second signature residue, Ile, of the MI motif in the PglB protein constitute the Ser/Thr-binding pocket.

Based on the presence of the DK or MI motif, we classified the STT3/AglB/PglB proteins into two groups. All PglB and some AglB proteins contain the MI motif, whereas all STT3 and the remaining AglB proteins contain the DK motif or its variant type [15]. Thus, there are two types of Ser/Thr-binding pockets: the Lys-type and the Ile-type, according to the second signature residue in the DK/MI motif. Mutagenesis studies proved the essential roles of the second signature residue for the enzymatic activity. The substitution of the lysine residue with alanine in yeast STT3 resulted in a lethal phenotype [13] and the substitutions with seven different amino acid residues in *P. furiosus* AglB-L resulted in the reduction of the *in vitro* activity [12]. The replacement of the isoleucine residue by alanine also substantially decreased the *in vitro* activities of the *C. jejuni*[14] and *C. lari* PglBs [18].

The genome of the hyperthermophilic archaeon, *Archaeoglobus fulgidus*, encodes three AglB paralogous genes. We have named the AglB paralogs with a letter plus an optional number, such as L (long) or S1 (short, number 1). The long AglB (AF_0380) consists of 868 residues and is called *Af*AglB-L, and the other two short AglBs (AF_0329 and AF_0040) consist of 591 and 593 residues, and are called *Af*AglB-S1 and *Af*AglB-S2, respectively. *Af*AglB-S1 and *Af*AglB-S2 are the shortest among the currently known STT3/AglB/PglB proteins, and they share 68% sequence identity. In contrast, *Af*AglB-L only shares 25% identities with *Af*AglB-S1 and *Af*AglB-S2. It would be interesting to elucidate the distinct and complementary roles of the multiple OST enzymes in one organism. Mammalian cells have two STT3 paralogs, STT3A and STT3B, which form different OST isoforms with the other seven subunits. The STT3A-containing OST isoform is the central player in the co-translational N-glycosylation of the nascent polypeptide chains, and the STT3B-containing OST isoform mediates the co- and post-translational N-glycosylations of unmodified glycosylation sites missed by the STT3A-OST isoform [19]. The protozoan parasite *Trypanosoma brucei* has three STT3 paralogs, STT3A, STT3B, and STT3C, and the three STT3 proteins constitute the three single-subunit OST enzymes. These enzymes have different specificities for the oligosaccharide moieties of the LLO donors and peptide acceptor sites [20]. For example, STT3A has stricter specificity for a particular type of lipid-linked oligosaccharide donor, and for glycosylation sites flanked by acidic residues, as compared to the other STT3 paralogs. In contrast, little is known about the different roles of the AglB paralogs in archaea.

We previously determined the crystal structures of the C-terminal globular domains of *Af*AglB-S1 and *Af*AglB-S2, at 1.75 and 1.94 Å resolutions, respectively [15,16]. In the present study, we determined the crystal structure of the C-terminal globular domain of *Af*AglB-L, as a fusion with maltose binding protein, at 1.90 Å resolutions. The three structures provide a complete overview of the three AglB paralogs encoded in the *A. fulgidus* genome. The information about their structural similarities and differences will be helpful to elucidate the distinct roles of the AglB paralogs not only in *A. fulgidus*, but also in other archaea.

## Results and discussion

### Crystallization of the C-terminal globular domain of *Af*AglB-L fused to maltose binding protein

The primary sequence of the full-length *Af*AglB-L protein consists of the N-terminal transmembrane region (499 residues) and the C-terminal globular domain (369 residues). First, we expressed the C-terminal globular domain of *Af*AglB-L in *E. coli* cells, and purified large quantities of the soluble protein. Although the protein was crystallized in a reproducible manner, the crystals only diffracted to low resolution. Next, we tried a fusion with *E. coli* K12 maltose binding protein (MBP), since successful examples of structure determinations using fusion proteins with MBP have been reported [21,22]. We connected MBP to the C-terminal globular domain of *Af*AglB-L, without a flexible linker sequence between them. This fusion protein is referred to as MBP-sAglB. We added a His tag at the N-terminus of MBP, for Ni-affinity chromatography. Amylose-affinity chromatography was not used for purification, because we wanted to test the apo and maltose-bound forms of MBP in the crystallization screening. In fact, MBP-sAglB crystallized only in the apo form in the absence of maltose, and the crystals diffracted to high resolution.

### MBP-sAglB forms a swapped dimer in the crystal

**Figure 1:**
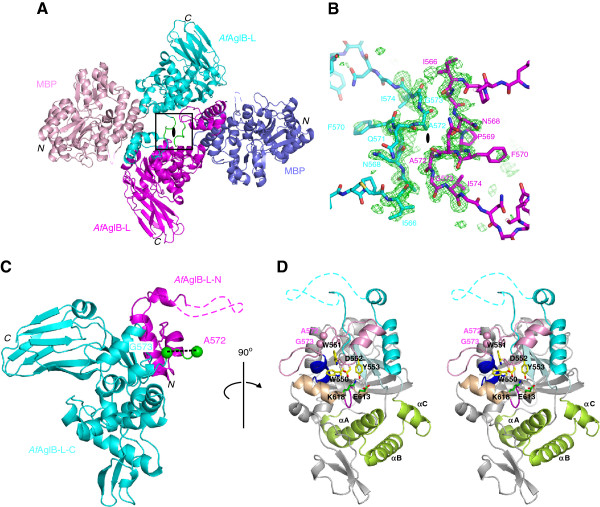
**Domain-swapped dimer structure in the crystal and reconstituted monomer structure of the MBP-sAglB fusion protein. (A)** Two MBP-sAglB molecules form a symmetric intertwined dimer, in which the molecules are related by a crystallographic 2-fold axis. The two molecules in the dimer structure are colored pink/magenta and blue/cyan. The hinge loop region is colored green. The dashed lines indicate the missing residues in the final model. **(B)** Close-up view of the hinge loop region (box, in **A**) with an Fo-Fc omit map contoured at +3σ. The simulated omit map was calculated by deleting residues 566–574, **(C)** Reconstituted monomeric structure of the C-terminal domain of *Af*AglB-L. The 73 residues from one molecule (*Af*AglB-L-N), and the 296 residues from another molecule (*Af*AglB-L-C), related by a 2-fold axis in the crystal, are colored magenta and cyan, respectively. The swapping site between Ala572 and Gly573 is shown as dashed lines between the two green spheres. **(D)** Stereo view of the reconstituted model of the C-terminal domain of *Af*AglB-L. The side chains of the WWDYG motif are labeled and colored yellow. The backbone of the WWDYG motif and following α-helix and loop are colored pink. The characteristic kinked helix is colored light brown, and the side chains of the first and second signature residues, E613 and K618, constituting the DK motif on the characteristic kinked helix are labeled and colored green. The amino acid sequence inserted in the DK motif is colored magenta. The cluster of three α-helices, are labeled as α_A_, α_B_, and α_C_, and colored yellow-green. The two pink spheres show the swapping site. The figures in **(C)** and **(D)** are related by a 90 degrees rotation about the vertical axis.

MBP-sAglB was crystallized in the monoclinic space group *C*2, with one monomer per asymmetric unit. The structure was solved to 1.90 Å resolution by the molecular replacement method using the structure of the maltose-free form of MBP [PDB: 1PEB] as the search model (Figure 1A). The positions of selenium atoms in the anomalous difference Fourier map calculated from the Se-SAD (selenium single-wavelength anomalous diffraction) data of the selenomethionine (SeMet)-substituted MBP-sAglB was helpful to interpret discontinuous electron densities corresponding to the C-terminal domain of *Af*AglB-L. The final model of MBP-sAglB was refined to R_*work*_ and R_*free*_ of 16.4% and 20.2%, respectively. Data collection and refinement statistics are listed in Table 1. In the final model, residues 171 to 180 in MBP were missing, as well as residues 524 to 540 in *Af*AglB-L. Interestingly, the C-terminal α-helix of MBP and the N-terminal α-helix of *Af*AglB-L were fused to form a long, continuous α-helical structure, which fixed the relative orientations of MBP and *Af*AglB-L in the crystal. This rigid connection may have facilitated the crystal growth. Apart from the direct covalent connection, the interactions between the C-terminal domain of *Af*AglB-L and MBP occurred but appeared minimal. The contact area was as small as 160 Å^2^, where two residues of MBP (K200 and K202) and two residues of *Af*AglB-L (W656 and D657) were involved. Thus, we concluded that no severe distortion was induced in the structure of the C-terminal domain of *Af*AglB-L by the extra noncovalent interactions within the fusion protein.

**Table 1:** Data collection and refinement statistics

	**Native**	**Se-SAD**
**Data collection statistics**^**a**^		
Beamline	SPring8 BL44XU	SPring8 BL44XU
Wavelength (Å)	0.9000	0.9790
Oscillation range (º)	180	1080
Space group	*C*2	*C*2
Cell parameters (Å)	*a* = 180.2, *b* = 52.9, *c* = 78.4	*a* = 181.6, *b* = 53.5, *c* = 79.0
Resolution range (Å)	50.0 – 1.90 (1.94 – 1.90)	50.0 – 2.30 (2.34 – 2.30)
Observed reflections	215,719	702,336
Unique reflections	58,464	32,931
Completeness (%)	99.5 (99.3)	99.8 (99.2)
*R*_merge_(*I*)^b^	0.071 (0.586)	0.175 (> 1)
*I* / σ(*I*)	25.0 (2.5)	16.3 (3.6)
**Refinement statistics**		
Resolution range (Å)	50.0 – 1.90	
No. of protein atoms	5,644	
No. of water molecules	553	
*R*_*work*_/*R*_free_^c^	0.164/0.202 (0.244/0.304)	
Rmsd^d^ from ideality bond length (Å)	0.007	
angles (º)	1.1	
Ramachandran plot (%)^e^		
Favored region	98.3	
Allowed region	1.7	
Outlier region	0.0	

^a^Values in parentheses are for the highest resolution shell.

^b^*R*_merge_(*I*) = (ΣΣ|*I*_i_ - <*I* > |)/ΣΣ*I*_i_, where *I*_i_ is the intensity of the *i*th observation and <*I* > is the mean intensity.

^c^*R*_work_/*R*_free_ = ∑|*F*_O_ - *F*_C_|/∑|*F*_O_|. *R*_work_ was calculated from the working set (95% of the total reflections). *R*_free_ was calculated from the test set, using 5% of the total reflections. The test set was not used in refinement.

^d^rmsd, root mean square deviation.

^e^Calculated using the program RAMPAGE.

**Figure 2:**
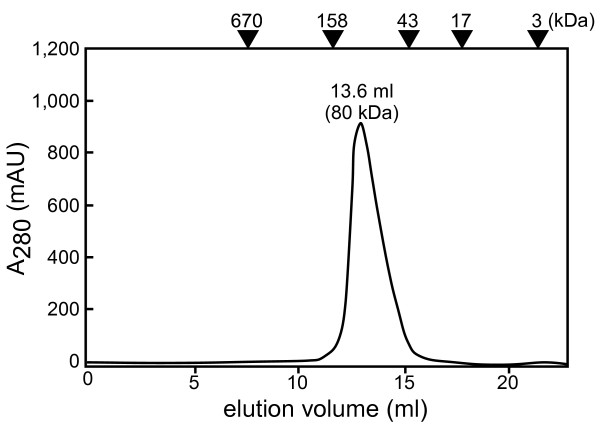
**Gel filtration chromatography elution profile.** The positions of the molecular weight markers are shown. The MBP-sAglB protein was eluted at the monomer position (experimental and theoretical molecular masses are 80 kDa and 85 kDa, respectively).

We found that the two molecules of MBP-sAglB exhibited an intertwined structure, related by a 2-fold rotational axis in the crystal (Figure 1A). This is not surprising, because there are many examples of intertwined structures in crystals [23,24]. The hinge loop region is defined as the segment that links the swapped segment to the rest of the protein. We found that the Asn568-Pro-Phe-Gln-Ala-Gly573 segment in the *Af*AglB-L portion was the hinge loop region (Figure 1B). The MBP-sAglB protein eluted as a monomer (ca. 80 kDa) in gel filtration chromatography (Figure 2). Thus, the formation of the intertwined dimer is a crystallographic artifact and lacks biological significance. We created a monomeric image of the C-terminal globular domain of *Af*AglB-L, by restoring the swapped segment (Figure 1C and D). The swapping was performed between A572 and G573, and the restored molecule consists of 73 residues (residues 500–572, magenta) from one molecule, and 296 residues (residues 573–868, cyan) from another molecule related by a 2-fold axis in the crystal.

### Overall structure of the C-terminal globular domain of *Af*AglB-L

**Figure 3:**
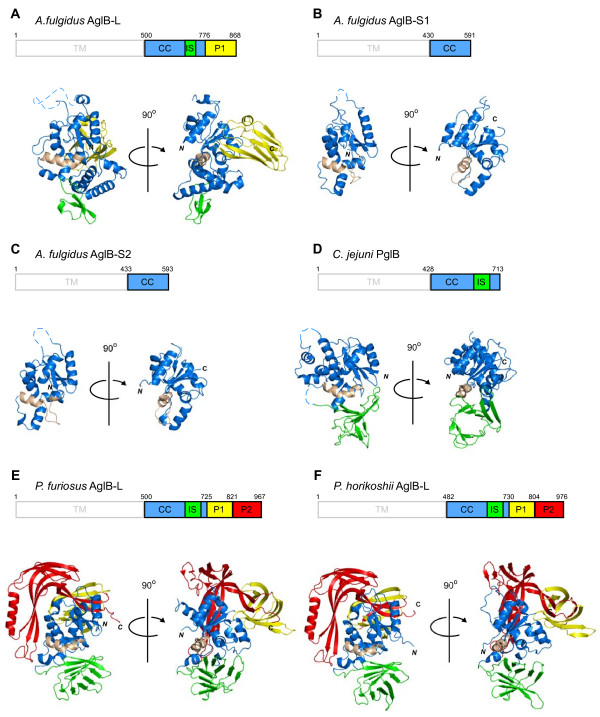
**Structural comparison of the C-terminal globular domain of *Archaeoglobus fulgidus *AglB-L with other archaeal and eubacterial structures. (A)***A. fulgidus* AglB-L, **(B)***A. fulgidus* AglB-S1, **(C)***A. fulgidus* AglB-S2, **(D)***Campylobacter jejuni* PglB, **(E)***Pyrococcus furiosus* AglB-L, and **(F)***Pyrococcus horikoshii* AglB-L. The domain organization is shown: TM, transmembrane; CC, central core; IS, insertion; P1, peripheral 1; P2, peripheral 2. The TM region, which was not included in the structure determination, is outlined in gray. The CC unit is colored blue, IS is green, P1 is yellow, and P2 is red. The characteristic kinked helix in the CC unit is colored light brown, as a landmark for comparison.

The overall structure of the C-terminal domain of *Af*AglB-L consists of three structural units, called CC (*c*entral *c*ore), IS (*i*n*s*ertion), and P1 (*p*eripheral *1*) (Figure 3A). We compared the new structure with the previously determined structures (Figure 3B-F): *Af*AglB-S1, *Af*AglB-S2, *Campylobacter jejuni* PglB (*Cj*PglB), *Pyrococcus furiosus* AglB-L (*Pf*AglB-L), and *P. horikoshii* AglB-L (*Ph*AglB-L). In addition to the common structural unit CC (*blue*), the C-terminal domains of the most AglB/PglB contain additional unique structural units, IS (*green*), P1 (*yellow*), and/or P2 (*red*). In contrast, *Af*AglB-S1 and *Af*AglB-S2 only consists of the CC unit, indicating the indispensable role of the CC unit for the catalytic activity of the OST enzyme. In accordance with this notion, the CC units of the six structures share overall structural similarity. The CC unit features a mixed α/β fold, and contains the conserved WWDYG and DK/MI motifs (Figure 1D). The length of the CC unit of *Af*AglB-L (243 residues) is longer than those of the other five AglB/PglB proteins (152–181 residues). The additional sequence forms three α-helices (α_A_, α_B_, and α_C_), which is unique among the six structures (Figure 1D). The IS unit is referred to as an insertion, because it seemed to be inserted into the amino acid sequence of the CC unit. The IS unit is a 9-stranded β-barrel-like structure in *Pf*AglB-L, *Ph*AglB-L, and *Cj*PglB, but in *Af*AglB-L, it is smaller and differs from the β-barrel-like structure. The unique cluster of the three α-helices in the CC unit appears to substitute for the small IS unit in the *Af*AglB-L structure. The P1 unit of *Af*AglB-L is β-sheet rich and occupies a similar spatial position to those in *Pf*AglB-L and *Ph*AglB-L, but the arrangement of the β-strands is also different. Thus, the other structural units besides the CC unit may have special roles in each OST enzyme. For example, they may contribute toward the increased thermal stability of the AglB proteins in the hyperthermophilic archaea, *Archaeoglobus* and *Pyrococcus*.

### Kinked helix with a short insertion sequence

**Figure 4:**
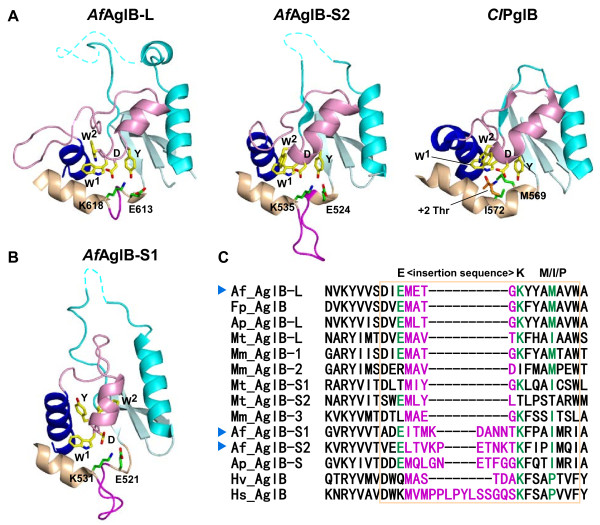
**Structural comparison of the Ser/Thr-binding pockets. (A)** Close-up views of the Ser/Thr-binding pockets of *A. fulgidus* AglB-L and AglB-S2. The Ser/Thr pocket of *C. lari* PglB in the complex with the Thr residue in the glycosylation sequon is shown for comparison. The conformations of the Ser/Thr pockets are similar to each other, and represent the resting state and the peptide substrate-bound state. The same color code is used as in Figure 1. **(B)** Close-up view of the Ser/Thr-binding pocket of *A. fulgidus* AglB-S1. The structure of the Ser/Thr-binding pocket is distorted, due crystal packing effects or the lack of interactions with the TM region. **(C)** Multiple sequence alignment of the variant types of DK motifs found in the classes *Halobacteria*, *Archaeoglobi*, and *Methanomicrobia*. The alignment was performed with the program MAFFT, without manual adjustment. The three signature residues of the variant types of DK motifs are highlighted in green. The inserted loop sequences are shown in magenta. The region of the characteristic, kinked helices is enclosed in the light-brown box. The following AglB sequences were used: Af_AglB-L [UniProt/TrEMBL: O29867_ARCFU], Af_AglB-S1 [029918_ARCFU], Af_AglB-S2 [O30195_ARCFU], Ap_AglB-L [D2RHL5_ARCPA], Ap_AglB-S [D2RDQ2_ARCPA], Fp_AglB [D3S254_FERPA], Mm_AglB-1 [Q8PZ47_METMA], Mm_AglB-2 [Q8PZ48_METMA], Mm_AglB-3 [Q8PUW8_METMA], Mt_AglB-L [A0B996_METTP], Mt_AglB-S1 [A0B8C2_METTP], Mt_AglB-S2 [A0B9E5_METTP], Hv_AglB [A9JPF0_HALVO], and Hs_AglB [Q9HQP2_HALSA]. The sequences of the three paralogous AglB proteins from *Archaeoglobus fulgidus* are marked with blue arrowheads.

The DK/MI motif resides on the characteristic kinked helix in the CC structural unit (Figure 3). The kinked helix consists of the N-terminal α-helical half and the C-terminal 3_10_-helical half. In our previous studies, we found that the *Af*AglB-S1 and *Af*AglB-S2 structures both contained an insertion sequence at the junction site of the two helical structures (Figure 4A and B). We concluded that the DK/MI motif of the two *Af*AglB proteins was a variant type of the DK motif with an insertion. Since this unexpected insertion separated the first and second signature residues of the DK motif in the primary structure, the identification of the variant type of DK motif would have been almost impossible without reference to the three-dimensional structures (Figure 4C). The consensus sequence of the variant type of DK motif was defined as E<>K*XXX*(M/I/P), where <> denotes the inserted sequence with a variable length [15]. In spite of the existence of abundant information, we could not clearly identify the DK/MI motif of *Af*AglB-L, due to the presence of redundant acidic residues in this region. By reference to the present *Af*AglB-L structure, we concluded that the kinked helix of *Af*AglB-L contained a four-residue insertion between the first and second signature residues, Glu613 and Lys618, respectively, of the variant type of DK motif. The spatial arrangement of the signature residues in *Af*AglB-L is identical to those in *Af*AglB-S1 and *Af*AglB-S2 (green side chains in Figure 4A and B).

### The Ser/Thr-binding pocket in various AglB and PglB structures

We focused our attention on the Ser/Thr-binding pocket in the CC unit. The Ser/Thr-binding pocket was first identified in *C. lari* PglB, in a complex with a substrate peptide (Figure 4A). The canonical structure of the Ser/Thr pocket was also found in the previously determined *Af*AglB-S2 structure and the present *Af*AglB-L structure (Figure 4A). Since both of the *Archaeoglobus* AglB proteins were crystallized in the absence of peptide substrates, we concluded that the Ser/Thr pocket was formed prior to peptide binding. The PglB protein contains the MI motif, whereas the two *Archaeoglobus* AglB proteins contain the DK motif. Thus, we also concluded that the Ser/Thr-binding pocket is a functional structure present in all of the OST enzymes, independently of the DK or MI motif.

In contrast, *Af*AglB-S1 has a deformed structure of the Ser/Thr-binding pocket [15]. The side chain of the tyrosine residue in the WWDYG motif protrudes in a different direction, and the α-helix following the WWDYG motif (*pink*) is also oriented differently (Figure 4B). Indeed, we also found considerable conformational variation of the WWDYG motif in *Pf*AglB-L and *Ph*AglB-L [16]. We inferred that this phenomenon suggested the remarkable plasticity of the WWDYG motif, and hence the flexibility of the Ser/Thr pocket. Indeed, the dynamic nature of the WWDYG motif and the following α-helix was confirmed in an NMR relaxation study of the C-terminal domain of *Af*AglB-S2 in the absence of substrates [16]. We speculate that the transient collapse of the Ser/Thr pocket must occur during the catalytic cycle, although the Ser/Thr pocket in the C-terminal domain has a canonical structure in the resting state and a peptide-bound state, as represented by *Af*AglB-L, *Af*AglB-S2, and *Cj*PglB, in the absence, and by *Cl*PglB, in the presence of a substrate acceptor peptide, respectively. The necessity of multiple conformational states in the enzymatic activity was suggested by a biochemical experiment using *Pf*AglB-L, in which the flexibility restriction forced by an engineered disulfide bond abolished the enzymatic activity, but its cleavage fully restored the activity [16]. Interestingly, in the crystal structure of MBP-sAglB, the domain swapping site is located in the segment corresponding to the flexible region identified in *Af*AglB-S2 [16].

## Conclusions

We have determined the crystal structure of the C-terminal globular domain of one of the three oligosac-charyltransferases in the hyperthermophilic archaeon, *Archaeoglobus fulgidus* (Figure 1). The crystallization of the fusion protein with MBP afforded high quality protein crystals. The C-terminal domain of *Af*AglB-L consists of three structural units, CC, IS, and P1 (Figure 3). Multiple sequence alignments in the region corresponding to the kinked helix in the CC unit were particularly difficult in the archaeal classes *Archaeoglobi*, *Halobacteria* and *Methanomicrobia*, due to the vast sequence diversity and abundance of acidic residues. The present *Af*AglB-L structure, together with the previously deter-mined *Af*AglB-S1 and *Af*AglB-S2 structures, provided the structure-guided sequence alignment, which indicated that all of the AglB paralogous proteins in these archaeal classes have the kinked helix, with inserted sequences of variable lengths (Figure 4C). The insertion sequences allow the spatial arrangement of the three signature residues of the variant type of DK motif to superimpose onto those of the canonical type of DK motif, found in STT3 and most AglBs (Figures 4A and B). The finding also supported a previously proposed rule: The catalytic subunits of the OST enzymes in one organism always contain the same type of DK/MI motif, and thus the same type (either the Ile-type or Lys-type) of Ser/Thr-binding pocket [15]. This information will be useful to understand the distinct and complementary roles of the two to four STT3/AglB paralogs coexisting in one organism.

## Methods

### Protein expression and purification

The DNA sequence of the C-terminal globular domain (residues 500–868) of *Af*AglB-L [UniProt/TrEMBL: O29867_ARCFU, AF_0380] was amplified by PCR from the genomic DNA, and that of maltose binding protein (MBP, residues 1–366) was amplified from the plasmid pMAL-c5x (New England Biolabs). The two DNA fragments were combined by the SOEing PCR method [25]. The final PCR product was cloned into *Sma*I-*Xho*I digested pET-47b (Novagen), using an In-Fusion Advantage PCR Cloning Kit (Clontech). The resultant fusion protein contained an N-terminal His_6_ tag. The expression plasmid was transformed into *E. coli* BL21 Gold (DE3) cells (Stratagene). The *E. coli* cells were grown at 310 K in LB media and selenomethionine core medium (Wako) for the production of native and SeMet derivative proteins, respectively, supplemented with 50 mg L^-1^ L-selenomethionine (Nacalai Tesque) and 30 mg L^-1^ kanamycin. When the A_600_ reached 0.6, isopropyl-1-thio-β-D-thiogalactopyranoside was added to a final concentration of 0.5 mM. After overnight induction at 289 K, the cells were harvested by centrifugation. The cell pellets were suspended in TS buffer (50 mM Tris buffer, pH 8.0, 100 mM NaCl) and disrupted by sonication. The recombinant protein was purified by affinity chromatography on nickel Sepharose High Performance resin (GE Healthcare), and the N-terminal His tag was removed by 3C protease, leaving a Gly-Pro-Gly extension at the N-terminus. The cleaved protein was further purified by gel filtration chromatography, using a Superdex200 10/300GL column (GE Healthcare) in TS buffer. The eluted protein was desalted and concentrated with an Amicon Ultra-15 centrifugal filter unit (Millipore, 100 kDa NMWL) to 40 mg mL^-1^ in 10 mM Tris–HCl buffer, pH 8.0, for crystallization.

### Crystallization

Initial crystallization screening was performed by the sitting drop vapor diffusion method, using the Index crystallization screen (Hampton Research), JCSG^+^Suite (Qiagen), PACT (Qiagen), and Classics (Qiagen) kits. After optimization, the native and SeMet crystals grew from a hanging drop with a 1:1 volume ratio (total volume, 2 μl) of the protein stock solution (40 mg mL^-1^, 10 mM Tris–HCl, pH 8.0) and the reservoir solution (0.1 M CAPSO buffer, pH 9.4, 33% polyethylene glycol 3350) at 293 K. Crystals were picked up with a nylon loop (Hampton Research), and then were directly cryo-cooled in liquid nitrogen. It was unnecessary to add any cryoprotectants, due to the high concentration of polyethylene glycol.

### Data collection, structure determination, and refinement

X-ray diffraction data were collected at beam line BL44XU of SPring-8 (Harima, Japan), and processed using the program *HKL2000*[26] to the resolutions of 1.90 Å and 2.30 Å for native and SeMet crystals, respectively. Molecular replacement and the phase improvement with solvent flattening were performed using the program *MR-Rosetta*[27]. The initial electron density map of the native data set obtained by the molecular replacement using the structure of the maltose-free form of MBP [PDB: 1PEB] as the search model showed discontinuous electron densities in the region corresponding to the C-terminal domain of *Af*AglB-L. Then, we calculated the electron density map using phases obtained by molecular replacement combined with SAD phasing, but the quality of the electron density map did not improve significantly. Further manual model rebuilding was performed with the program *COOT*[28], and subsequent crystallographic refinement was performed with the program *PHENIX*[29]. Fortunately, the positions of nine selenium atoms in selenomethiones in the C-terminal domain of *Af*AglB-L were clearly visible in the anomalous difference Fourier map calculated from the Se-SAD data set. The superposition of the coordinates of *Af*AglB-S1 [PDB: 3VGP] and *Af*AglB-S2 [PDB: 3VU0] onto the partially built model of the N-terminal α-helix of the C-terminal globular domain of *Af*AglB-L correctly placed the *Af*AglB-S1 and -S2 structures in the electron density maps. Because the CC unit is common in all AglB and PglB proteins, the superposed structures guided the manual model building and refinements to obtain the final model of the C-terminal domain of *Af*AglB-L to a resolution of 1.90 Å (Figure 1). The asymmetric unit contained one protein molecule. The calculated solvent content was 44.1% (*V*_M_ = 2.20 Å^3^ Da^-1^). Data collection and refinement statistics are summarized in Table 1. The atomic coordinates of MBP-sAglB have been deposited in the Protein Data Bank, with the accession code 3WAI.

The figures were generated with the PyMOL Molecular Graphics System, Version 1.3, (Schrödinger, LLC). The multiple sequence alignment was performed with the program *MAFFT*[30].

## Abbreviations

Agl: Archaeal glycosylation; CC: Central core; IS: Insertion; LLO: Lipid-linked oligosaccharide; MBP: Maltose binding protein; MBP-sAglB: Fusion protein of MBP and the C-terminal globular, soluble domain of *Archaeoglobus fulgidus* AglB-L; OST: Oligosaccharyltransferase; P1: Peripheral 1; P2: Peripheral 2; Pgl: Protein glycosylation; SAD: Single-wavelength anomalous diffraction; SeMet: Selenomethionine; STT: Staurosporine and temperature sensitivity; TM: Transmembrane.

## Competing interests

The authors declare no competing financial interest.

## Authors’ contributions

SM purified and crystallized the protein. SM and AS collected diffraction data, processed data, and solved and refined the structure. SM and DK designed experiments, evaluated results and wrote the manuscript. All authors read and approved the final manuscript.
